# Strategies for CRISPR-based knock-ins in primary human B cells and lymphoma cell lines

**DOI:** 10.3389/fimmu.2025.1589729

**Published:** 2025-05-16

**Authors:** Sophie Lund, Chun Gong, Xin Yu, Louis M. Staudt, Daniel J. Hodson, Sebastian Scheich

**Affiliations:** ^1^ Goethe University Frankfurt, University Hospital, Frankfurt am Main, Germany; ^2^ University Cancer Center (UCT) Frankfurt, University Hospital, Goethe University, Frankfurt am Main, Germany; ^3^ Frankfurt Cancer Institute, Goethe University, Frankfurt am Main, Germany; ^4^ Cambridge Stem Cell Institute, Cambridge Biomedical Campus, Cambridge, United Kingdom; ^5^ Lymphoid Malignancies Branch, National Cancer Institute, National Institutes of Health, Bethesda, MD, United States; ^6^ German Cancer Consortium (DKTK), Partner Site Frankfurt/Mainz, Frankfurt am Main, Germany

**Keywords:** gene editing (CRISPR/Cas9), knock-in, lymphoma, DLBCL, NF-kappa B (NF-KB)

## Abstract

Since its advent about ten years ago, the CRISPR-Cas9 system has been frequently used in biomedical applications. It has advanced various fields, and CRISPR-Cas9-based therapeutics have shown promising results in the treatment of specific hematological diseases. Furthermore, CRISPR gene editing technologies have revolutionized cancer research by enabling a broad range of genetic perturbations, including genetic knockouts and precise single nucleotide changes. This perspective focuses on the state-of-the-art methodology of CRISPR knock-ins to engineer immune cells. Since this technique relies on homology-directed repair (HDR) of double-strand breaks (DSBs) induced by the Cas9 enzyme, it can be used to introduce specific mutations into the target genome. Therefore, this methodology offers a valuable opportunity to functionally study specific mutations and to uncover their impacts not only on overall cell functions but also on the mechanisms behind cancer-related alterations in common signaling pathways. This article highlights CRISPR knock-in strategies, protocols, and applications in cancer and immune research, with a focus on diffuse large B cell lymphoma.

## 1 Introduction

Lymphomas are a group of heterogeneous malignancies that occur in B cells, T cells, and natural killer (NK) cells at various stages of maturation (1). Diffuse large B-cell lymphoma (DLBCL), which accounts for nearly 40% of all non-Hodgkin lymphoma diagnoses, is the most common type of lymphoma. However, DLBCL is an extremely heterogeneous disease with different responses to standard therapies, necessitating further research (2). Gene expression profiling has determined two major biological DLBCL subtypes, that resemble the gene expression of germinal center B cells (GCB) or activated B cells (ABC) (3). These subtypes reveal distinct oncogenic mechanisms: ABC DLBCL tumors rely on chronic, active B-cell receptor (BCR) signaling, which is driven by the clustering of BCR molecules on the cell surface (4). The BCR activates proximal kinases such as spleen tyrosine kinase (SYK) or Bruton’s Tyrosine Kinase (BTK) and the “CBM” multiprotein complex. The CBM complex consists of three proteins, CARD11, BCL10 and MALT1, that drives an oncogenic NF-κB signal together with a multiprotein complex - the My-T-BCR complex - consisting of the BCR, the immune adaptor protein MYD88 and Toll like receptor 9 (5). In contrast, the GCB subtype relies on tonic BCR signaling that is likely antigen independent but depends on the BCR coreceptor CD19, its associated cell surface protein CD81 and engages primarily PI3 kinase (PI3K)/AKT signaling (2).

Recent genetic classifications of DLBCL have further subdivided the ABC and GCB subtypes into distinct genetic subtypes (6–8). These subtypes are characterized by specific patterns of genetic aberrations. Understanding the functional impact of each of the many genetic variants is the critical next step to unlock the biology of these genetic subtypes. CRISPR/Cas9-based technologies allow us to model specific mutations by endogenous knock-ins in lymphoma cell lines and primary germinal center B cells and offer a powerful tool for the study of their functional mechanisms. This paper highlights strategies for CRISPR/Cas9-based knock-ins and their applications in lymphoma research.

## 2 The advancement of CRISPR/Cas9 as a cut-and-paste tool in genetic engineering

Studying specific mutations in preclinical lymphoma models is essential for understanding their functional mechanisms in lymphoma biology, signaling and lymphomagenesis. One approach that has revolutionized cancer research is to use the CRISPR/Cas9 system for genome editing (9). Early attempts to introduce mutations at precise sites and time points, rather than relying on random spontaneous mutations, began with studies of DNA damage and repair. The observation that double-strand breaks (DSBs) could be selectively introduced into the genome, then repaired by non-homologous end joining (NHEJ) with resultant local mutagenesis, provided the impetus for targeted genome editing. In 1987, the first clustered regularly interspaced short palindromic repeats (CRISPR) in the bacterial genome were discovered by Ishino et al. (10), and in further studies, the role of Cas proteins was elucidated (11). These were found to be rather abundant and diverse functions were described, like helicase, nuclease, or polymerase (12). Fifteen years after the first discovery of CRISPR-Cas9 in *E. coli* in 1987, George Church, Jennifer Doudna, Emmanuelle Charpentier, and Feng Zhang pioneered the use of this system as a “cut-and-paste” tool to specifically modify genomes (13) This forms the basis of our state-of-the-art methodology of CRISPR/Cas9-induced knock-ins.

## 3 CRISPR knock-in strategies and general considerations

Despite the challenges associated with CRISPR/Cas9-mediated knock-ins in B cells, this method remains a powerful tool for precise genetic modifications. While other methods like overexpression models seem to be a simpler way of investigating cancer-related alterations, they often hold many disadvantages such as significantly different expression levels compared to the endogenous state. Furthermore, this can lead to expression of artifacts, mis-localization, and altered functional responses (14). In addition, plasmid-based overexpression of genes mostly relies on synthetic promoters, thereby precluding studies of endogenously driven transcription (14). This means that overexpression experiments are a useful tool to investigate novel proteins involved in cellular processes (15), but when researching patient derived mutations and their impact on known pathways, it is of great advantage to use CRISPR/Cas9-based knock-in experiments for endogenous investigations.

Another key point to consider is that most gene editing is based on transient transfection, meaning that required modifications such as knock-down or overexpression, may only be harbored for a short time period (16). In contrast, knock-ins are a permanent gene modification and therefore a versatile tool in *in vitro* research, where long-term experiments are standard.

The ability to introduce targeted alterations further enables functional studies of oncogenic drivers and resistance mechanisms in lymphoma. Additionally, the system design is straightforward, facilitating quick customization for targeting various genes, expediting experimental timelines (17). Refining CRISPR knock-in strategies in B cells improves our ability to model disease-specific mutations. These advancements provide insights into lymphoma pathogenesis and contribute to the development of more personalized treatment approaches.

To achieve precise genome editing, it is essential to understand the key components of the CRISPR/Cas9 system. Its two main components are the tracrRNA:cRNA that is engineered as a single guide RNA (gRNA or sgRNA), a short synthetic RNA consisting of a double-stranded scaffold sequence for Cas-binding, and a user-defined 20 nucleotide spacer to set the target site by Watson-Crick base pairing (18). Additionally, the target needs to be immediately adjacent to a Protospacer Adjacent Motif (PAM), a 2–6 base pair motif, whose sequence functions as a binding signal for the Cas protein. The specific sequence depends here on the Cas protein of use, with the most common being SpCas9 derived from *Streptococcus pyogenes* and whose PAM is NGG (19). Once the Cas9 has bound to an appropriate PAM site, an R-loop is formed, resulting in RNA-DNA hybrid formation and thereby Cas9 activation. Then Cas9 induces a DSB, which is then repaired by the cellular machinery of the host (20). Two common repair mechanisms are non-homologous end joining (NHEJ) and homology-directed repair (HDR). Important to mention is that NHEJ takes place in the absence of exogenous homologous DNA and that it is a process that is active in all phases of the cell cycle (20). This makes NHEJ more prone to small random insertions or deletions (indels) at the cleavage site. In contrast, HDR based repair mechanisms are more precise, requiring the use of an exogenous homologous DNA template, that, in CRISPR-gene editing, contains the sequence of interest to be inserted (20).

CRISPR-mediated knock-ins have emerged as a powerful tool for studying B cell development and function by enabling precise genetic modifications that reveal key regulatory mechanisms in these immune cells. Nevertheless, having a genetically altered model system in B cells to investigate mutations in a translational manner has remained a challenge during the past years due to several factors. Thus, optimization strategies must be applied to the various components of the knock-in system, including enhancing HDR efficiency over NHEJ in DSB repair, designing the sgRNA, the PAM orientation, and the prevention of unwanted re-cutting. These factors will be further elucidated in the following section.

### 3.1 Enhancing HDR efficiency

B cells often reside in a quiescent state, favoring NHEJ over HDR (21, 22). As above mentioned, DSBs are induced by the Cas9 protein and can be either repaired by NHEJ or HDR. CRISPR/Cas9-mediated knock-ins rely on inserting foreign DNA sequences through HDR. Since NHEJ is active throughout the cell cycle, strategies to enhance HDR mediated repair or suppress NHEJ are key. Thus, optimizing HDR efficiency should be considered before initiating a CRISPR knock-in experiment (23). An efficient way to do this is through the HDR template design. It is known that strand preference and optimal homology arm lengths are important when designing an HDR template. Therefore, it is recommended to have 30–60 nt lengths for the homology arms if using short donor oligos. For longer HDR donors, 200–300 nt lengths are recommended (17). When the insertion is placed in close proximity to the cut site, there is no preference for targeting versus non-targeting strand described. However, when the edit takes place outside the recommended 5–10 bp distance from the cut site, specific strand preferences need to be taken into account. Previous research has shown that the targeting strand, the strand where the Cas9 protein binds, is preferred for PAM-proximal edits, while the non-targeting strand shows benefits for PAM-distal edits (17). In addition to that, also the length of insertion needs to be considered, since it was observed that it has an influence on whether single stranded or double stranded HDR templates are favored. While for small insertions (such as FLAG-tags or HIS-tags, or for missense mutations) single stranded DNA represents the best HDR, for larger inserts like fluorescent proteins or degron tags, single stranded templates are less efficient. Here, a small plasmid including the template flanked by 500 nt homology arms is a preferred template, which contains the fluorescent protein/degron tag linked to a 2A linker, which can be electroporated into the cells along with the Cas9-sgRNA ribonucleoprotein (RNP) complex.

Another approach to enhance HDR mediated repair is the use of small molecule inhibitors that suppress NHEJ. A variety of small molecules have been tested for this purposes such as reomidepsin or nedisertib (24, 25). Several companies sell their own, proprietary compounds to enhance HDR repair.

### 3.2 sgRNA design

The design of the required sgRNA influences the knock-in efficiency as well. Factors impacting sgRNA efficacy include GC content and melting temperatures, as well as cutting frequencies, which reflects how effectively Cas9 cuts at the target site while minimizing off-target effects (23). The closer the targeted genomic alteration is to the Cas9 cutting site, the higher the efficiency of the respective knock-in (17). Several tools can aid sgRNA design by considering all these factors to predict the most suitable sgRNA, its binding site, as well as on- and off-target efficiencies. In our hands in B cell models, distances up to 10 base pairs are tolerable, however, if the cut site is further than 10 base pairs away from the desired genomic edit, efficiency drops dramatically.

### 3.3 PAM orientation

PAM orientation varies across different CRISPR/Cas systems. In some Cas systems, the PAM is positioned on the same strand that base pairs with the sgRNA. However, Cas9 typically follows a guide-centric orientation, with the PAM located on the strand that matches the sgRNA (26).

### 3.4 Prevention of unwanted re-cutting

Once the desired edit is introduced, cutting of the target locus must be terminated. The introduction of silent mutations into the protospacer or PAM sequence, which can be chosen from a set of mutations following empirically defined rules to provide the highest HDR rates, prevents continued, unwanted re-cutting at the same locus after the repair (17). Similar to the above mentioned strand preferences regarding the cut site, PAM orientation should also be considered when incorporating silent mutations. For PAM-distal HDR edits, the non-targeting strand containing silent mutations placed within the protospacer sequence is preferred and vice versa (17). If it is not possible to introduce a silent PAM mutation, at least two silent mutations in the sgRNA binding region should be created to prevent re-cutting after successful genomic edit.

## 4 Practical considerations for knock-in strategies in human B cells and cell lines

The efficient delivery of donor DNA into B cells harbors challenges due to their resistance to common transfection methods like lipid transfection, electroporation, and nucleofection, even though the latter remains more feasible (27). Thus, several methods of Cas9 and sgRNA delivery into the cell were tested previously to evaluate the on-target DNA cutting efficiencies, of which the delivery via *in vitro* performed ribonucleoprotein (RNP) complex showed the best results (23). The following considerations are optimized for the NEON electroporation system (28), where the same buffers can be used for different cell types, but the instrument settings for electroporation need to be optimized for the specific cell line in use. Another commonly used system is the Amaxa 4D Nucleofector System from LONZA (29), which uses a slightly different protocol, but the optimization strategies remain the same. Before starting the experiment, one must determine which electroporation system is the most suitable one since they can be adapted for different needs.

To form the RNP complex,0.5 µl sgRNA (44 µM) are combined with 0.5 µl Cas9 protein (37.2 µM), incubated for 20 minutes at room temperature and then mixed with 1.5 µl HDR template (33.3 µM) and 2 µl electroporation enhancer (10.8 µM). While incubating the RNP, a 24-well plate can be prepared with 1 ml growth medium and 1.5 µl HDR enhancer (e.g. IDT ALT-R- HDR Enhancer) per well and placed in a 37 C° incubator until electroporation. Then 0.5x10^6^ target cells are collected, washed with 1xPBS, and resuspended in 8.5 µl Buffer R (provided in the NEON electroporation kit (28)).

Then, the total of 4.5 µl RNP/HDR mixture is combined with 8.5 µl cells, and mixed well by pipetting, avoiding air bubbles. The NEON electroporation cartridge (28) is then filled with 3 ml Buffer E, 10 µl of the cell mix aspirated slowly with the NEON electroporation pipettor and placed into the cartridge. On the electroporation device, settings such as voltage, pulse number, and duration can be selected depending on the cell line in use.

Important to mention is that the growth medium must be replaced 24 and 48 hours after electroporation since the HDR enhancer, electroporation buffers, and extra DNA/RNA pieces carried from electroporation can be toxic to the cells.

To confirm the incorporation of the desired mutation, pooled cells should be lysed using a Tris/EDTA buffer supplemented with ProteinaseK and RNaseA. The resulting DNA can be used in a PCR reaction using high-fidelity polymerase and analyzed by Sanger sequencing. This verification step is typically performed after approximately one week of cell growth, though the timing may vary depending on the cell type. Another strategy to validate knock-in efficiency is to introduce additional restriction enzyme digestion sites by adding silent mutations. Then, the PCR product can be digested with the respective enzyme and the digested versus non-digested band quantified on a gel. This is especially helpful when knock-in efficiencies are low, making the mutation hard to detect by Sanger sequencing of a pooled population.

**Figure 1:**
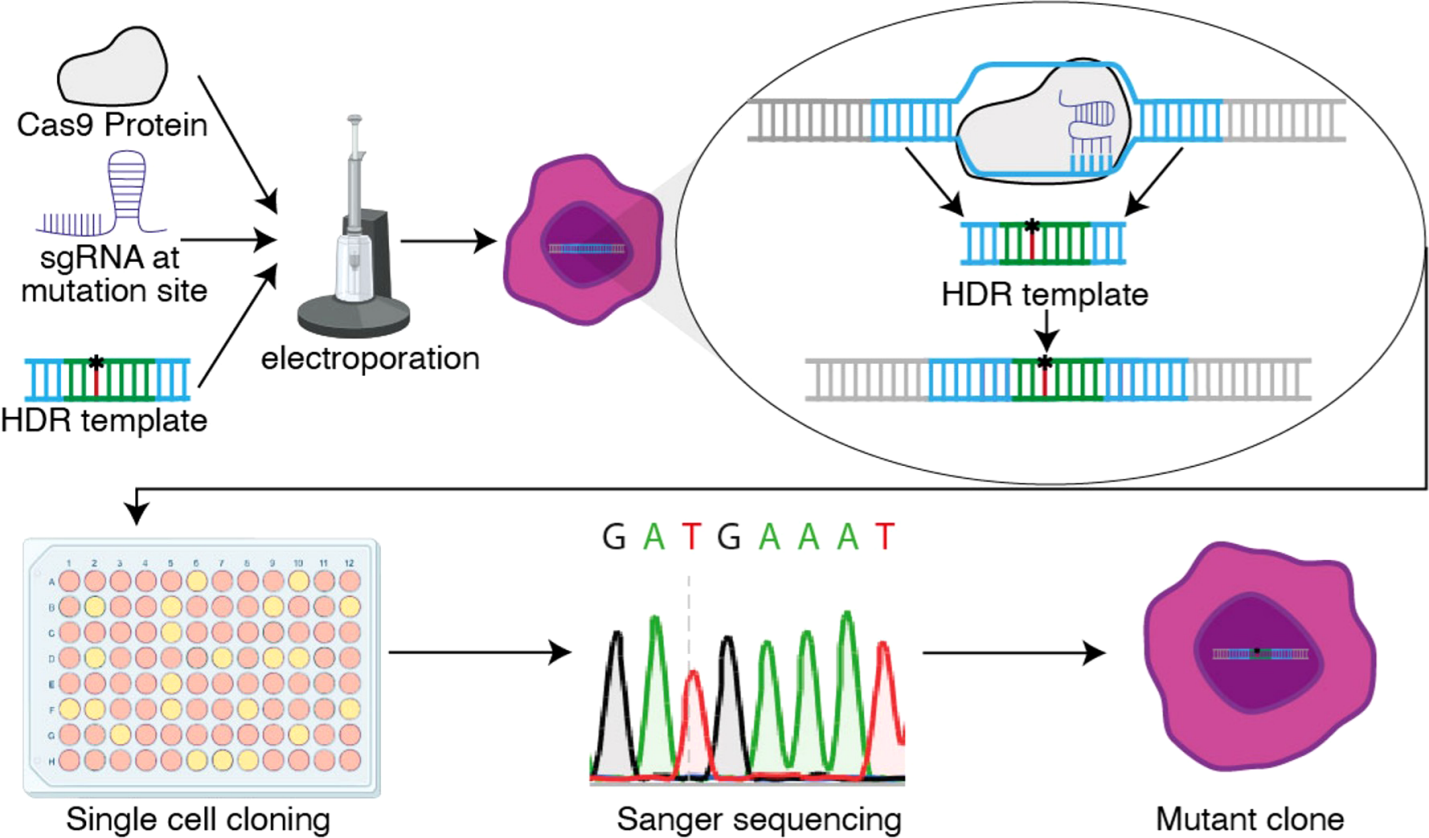
Schematic overview of the CRISPR knock-in protocol. The Cas9 protein, sgRNA and HDR template enter the cell via electroporation, where the HDR template is replacing the original DNA sequence through homology directed repair (HDR). After successful electroporation, single cells are plated via limiting dilution and Sanger sequenced to determine the clones with a homozygous knock-in.

The pooled population of cells will contain a mix of edited and unedited cells. Moreover, mutations may be heterozygous or homozygous. To generate clones that contain a homozygous knock-in, cells should be plated for clonal selection. This can be achieved via single cell fluorescence-activated cell sorting (FACS) or by limiting dilution, plating approximately 20–50 cells per plate in a 96-well. After two weeks of growth of these single cell clones, sequencing should confirm homozygous or heterozygous knock-in. This approach is summarized in **Figure 1**.

## 5 BCL10 mutation as a model for altered NF-κB signaling

**Figure 2:**
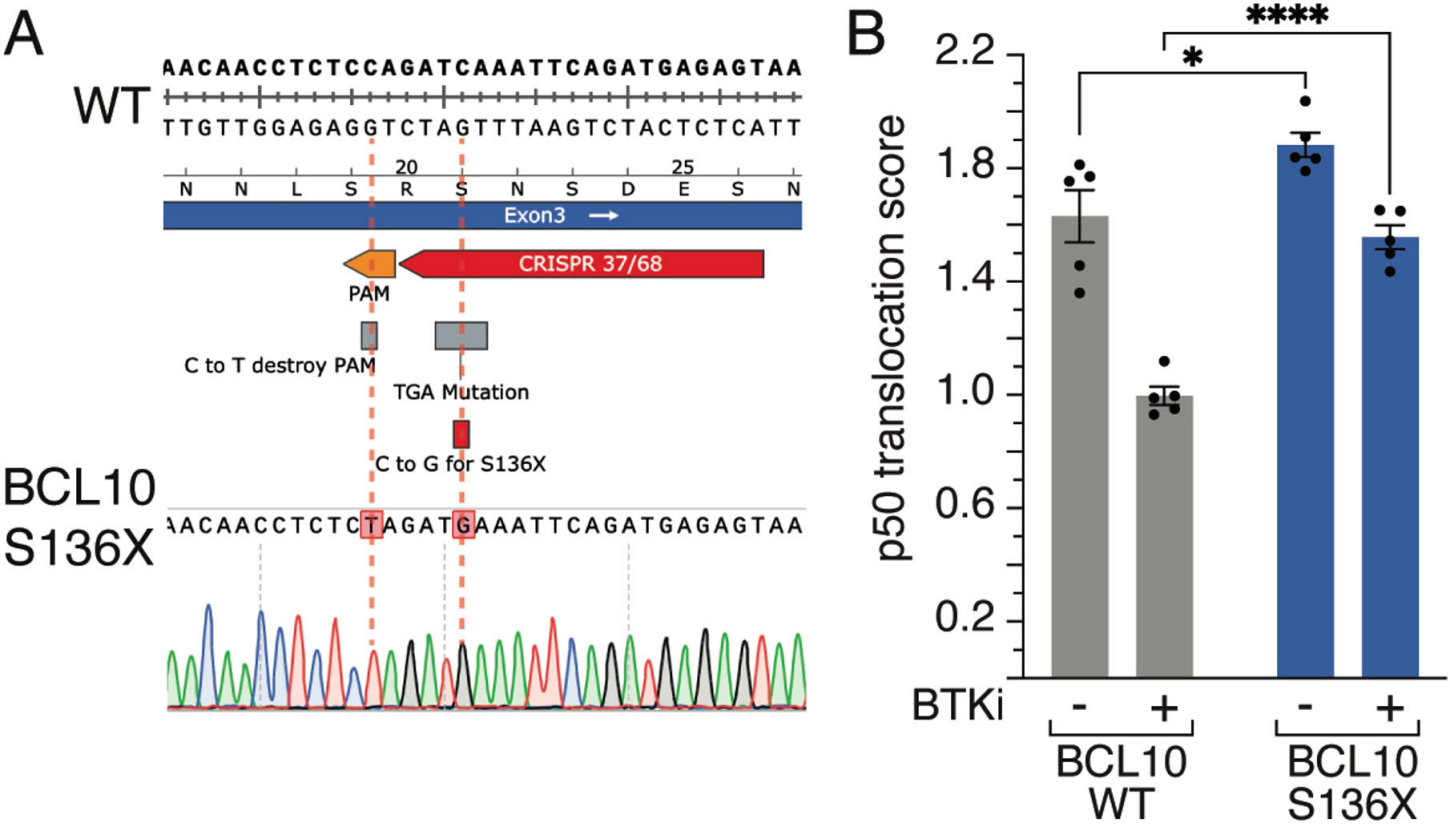
BCL10 mutations. **(A)** Sanger sequencing result of a BCL10 mutation. The cytosine at position 136 is replaced by a guanine, resulting in the S136X mutation. **(B)** Mean nuclear NF-κB (p50) translocation score assessed by ImageStream flow cytometry in wildtype (WT) or BCL10 S136X mutant TMD8 cells treated for 16 hours with DMSO or acalabrutinib. *, P ≤ 0.05, ****, P ≤ 0.0001 (one-way ANOVA). Data from 5 independent replicates. Error bars represent SEM.

One example of a gene frequently mutated in DLBCL is BCL10. BCL10 plays a crucial role in lymphoma through its involvement in the NF-κB signaling pathway, which is essential for B cell survival and proliferation. As a key component of the CBM complex, alongside MALT1 and CARD11, BCL10 facilitates NF-κB activation, driving transcription of pro-survival and pro-inflammatory genes (30, 31). Especially in ABC-DLBCLs, recurrent somatic mutations of BCL10 lead to chronic activation of the CBM complex and thus NF-κB activation (6, 7). These mutations can be divided into two functionally distinct groups, either missense mutations of the BCL10 CARD domain or truncation of its C-terminal tail (30), which has been demonstrated using overexpression models. We generated a BCL10 S136X mutation following the methodology described in this paper with a PAM mutation. We changed a cytosine to a guanine to create the S136X mutation and validated single clones by Sanger sequencing (**Figure 2A**). Next, we measured using imaging flow cytometry the nuclear translocation of the active NF-κB subunit p50 after treatment with the BTK inhibitor (BTKi) acalabrutinib. We observed a reduction of p50 nuclear translocation upon BTKi, however, this effect was rescued in cells harboring the endogenous BCL10 S136X mutation (**Figure 2B**). This data further highlights the importance of BCL10 mutations in addition to published literature (30) particularly because BCL overexpression is associated with a strong growth advantage, and we demonstrate here a functional role for BCL10 mutations in the endogenous setting. Furthermore, this example underlines the importance of knock-ins to functionally study lymphoma biology.

## Data Availability

The original contributions presented in the study are included in the article/Supplementary Material. Further inquiries can be directed to the corresponding author.
